# The experience and impact of living with idiopathic hypersomnia: A qualitative study of patient perspectives shared in online media

**DOI:** 10.1371/journal.pone.0333497

**Published:** 2025-10-22

**Authors:** Sarah L. Bermingham, Alexander Spalding, Elisabeth Bennett, David T. Plante

**Affiliations:** 1 Takeda Pharmaceuticals USA, Cambridge, Massachusetts, United States of America; 2 Gemic, Berlin, Germany; 3 Gemic, Toronto, Ontario, Canada; 4 Department of Psychiatry, University of Wisconsin-Madison, Madison, Wisconsin, United States of America; Shahrood University of Medical Sciences, IRAN, ISLAMIC REPUBLIC OF

## Abstract

**Introduction:**

Understanding the experience and impact of idiopathic hypersomnia (IH) is critical for improving diagnosis, treatment, research, drug development, and policy awareness. Currently, physician and researcher perspectives dominate the discourse on IH, often overlooking the lived experiences of those with the condition.

**Objective:**

To understand the lived experience of IH from the patient perspective by analyzing spontaneously generated online patient-experience data.

**Methods:**

Publicly available data were collected through iterative Google searches, manually coded, and thematically analyzed using inductive and deductive approaches to qualitative content analysis. Concept saturation was reached, ensuring comprehensive theme exploration.

**Results:**

Searches identified 346 social media posts, community forums, blogs, videos, and podcasts created by 123 people with self-identified IH between 2012 and 2022. Most were female, 16–60 years old, and lived in the United States, Australia, Europe, and Canada. Symptom experiences were grouped under 10 themes: prolonged sleep, never feeling fully awake, relentless sleepiness, non-restorative sleep, difficulty in waking, automatic behavior, microsleeps and prolonged naps, cognitive difficulties, limited physical energy, and vivid dreams and hypnogogic hallucinations. Life impacts were identified and grouped under seven domains: psychological and emotional well-being, activities of daily living, injuries, relationships, work and school, physical health, and healthcare and treatment burden.

**Discussion:**

This study expands the understanding of IH beyond clinician and researcher-driven perspectives and clinical descriptions and illuminates its profound impact on all aspects of life from patients’ point of view. These insights can help clinicians provide better care, drive patient-centered drug development, and raise awareness of this devastating disorder.

## Introduction

Idiopathic hypersomnia (IH) is a chronic sleep disorder of suspected neurological origin, although the underlying cause remains unknown [1]. People with IH (PwIH) experience excessive daytime sleepiness, prolonged nocturnal sleep, severe sleep inertia, long and unrefreshing naps, profound fatigue, cognitive difficulties, autonomic dysfunction, and anxiety and depression [1,2]. Two clinical subtypes were historically recognized: IH with long sleep time (e.g., ≥ 10 hours per 24 hours) and IH without long sleep time [2]. However, the International Classification of Sleep Disorders, Third Edition, Text Revision now classifies IH as a single central disorder of hypersomnolence [3]. IH is considered a rare disorder, with prevalence estimates ranging from 0.5 to 37.7 per 100,000 people, depending on the criteria used to define cases [4–8].

IH has a profound impact on quality of life, significantly impairing emotional wellbeing, daily functioning, work productivity, career choices, social participation, and personal relationships [9,10]. These impacts extend beyond the individual, affecting the lives of partners, family members, and society [11,12]. Treatment options are limited. Currently, the only medication approved for IH, a nighttime oxybate, is available exclusively in the United States [13]. Other treatments, such as stimulants and wake-promoting agents approved for narcolepsy, are used off-label [14,15]. PwIH often cycle through multiple medications, facing challenges such as inadequate efficacy, poor tolerability, and numerous access barriers.

Most of what is known about IH has been shaped by clinical observations and researcher-developed surveys. Symptom definitions are largely informed by healthcare professionals and applied uniformly across sleep disorders, despite meaningful differences in how symptoms are experienced. For example, while people with narcolepsy report an episodic, overwhelming need to sleep that is relieved by naps, PwIH describe a relentless, pervasive sleepiness throughout the day that is not alleviated by sleep [16,17]. Yet, both experiences are labeled “excessive daytime sleepiness”, highlighting the need for a more nuanced understanding of symptom manifestation. Similarly, questionnaires used to assess impairment in PwIH were primarily designed based on research involving people with narcolepsy and the clinical experiences of investigators [9,11,18–24]. Given the distinct symptomatology of IH compared with narcolepsy, and differences in perspectives between clinicians and patients, these studies may neglect crucial aspects of IH impact. Moreover, quantitative surveys cannot reveal the causes of peoples’ limitations, nor help others comprehend the seriousness and severity of this disorder.

First-person perspectives of PwIH are largely absent from academic literature. The most meaningful efforts to center the patient voice have come from advocacy-led initiatives, such as the *Voice of the Patient* report developed through the U.S. FDA’s patient-focused drug development program [25]. Drawing on patient testimonies and survey data, the report highlights several challenges faced by PwIH, including diagnostic delays, inadequate treatments, and stigma, and emphasizes the urgent need to capture the full burden of IH from patient perspective. These insights are essential for improving diagnosis, developing more effective treatments, addressing unmet needs, quantifying the costs of medications, creating patient-relevant outcome measures, and better engaging providers and policymakers, who often lack awareness of IH [26,27].

Qualitative research offers a powerful approach for capturing the richness and complexity of patient experiences. However, traditional approaches, such as semi-structured patient interviews or focus groups, are typically guided by a flexible discussion protocol containing topic areas, questions, and prompts created by researchers [28]. In conditions like IH, where the full scope of the patient experience is not yet well understood, these methods may unintentionally reproduce existing assumptions and constrain the emergence of novel insights. In contrast, exploring what PwIH spontaneously express about their condition can offer unfiltered insights to improve understanding and guide future research.

Online platforms and smartphone use have increasingly enabled this spontaneous expression in recent years. Individuals with health conditions can share, learn, teach, and support one another, and the most active communities are those with long-term conditions and rare diseases [29,30]. People on these platforms create vast amounts of spontaneously generated online patient-experience (SGOPE) data, including unstructured text, video, audio, and artwork [31]. Studies across many therapeutic areas have used SGOPE data to investigate disease burden [32,33], and the US Food and Drug Administration encourages using social media as a preliminary source of patient-experience data to support patient-focused drug development [34].

This study aims to provide a comprehensive understanding of the symptom experiences and impacts of IH from the perspective of those affected by analyzing SGOPE content to identify common key themes and patterns. This research seeks to offer insights that will improve clinical understanding and inform patient-centered research.

## Methods

### Study design

This study was a qualitative analysis of publicly available SGOPE content created by people who self-identified as living with IH. Content was manually collected and analyzed using inductive and deductive approaches [35]. The research was conducted in compliance with the terms and conditions of each platform.

### Search strategy

A purposive sampling approach was used to search for publicly available SGOPE content related to IH. Google searches were conducted using initial search terms (“idiopathic hypersomnia” or “IH,” combined with “patient story,” “my story,” “feelings,” “experience,” “impact,” “challenges,” or “burden”) to capture a broad range of content across social media platforms (Discord, Facebook, Instagram, Reddit, TikTok, YouTube, X/Twitter), advocacy websites, blogs, news outlets, and podcasts. As themes emerged, additional search terms such as “stigma,” “well-being,” “social,” “family,” “friends,” “career,” “quality of life,” “mental health,” “physical,” “sensory,” “eating,” “exercise,” “self-care,” and “treatment” were introduced to capture more specific aspects of IH. Searches were conducted iteratively between September 27, 2022, and October 21, 2022, until concept saturation was reached.

### Data selection

English-language content was included if the authors self-identified as having IH using phrases like “I was diagnosed with IH,” “I have IH,” or “My IH,” or if content was published on curated platforms such as patient advocacy websites, and the content contained rich data on the experience and impact of IH. Richness was subjectively assessed based on the dimensionality of the experiences described and the depth of insight offered. Longer content was generally considered richer, although shorter sources (e.g., TikTok videos) were included if they addressed analytically relevant aspects of IH. There was no limit on the number of posts collected from each individual.

### Data extraction

Text, video, and audio posts were transcribed verbatim, and descriptions were created for silent videos and illustrations. Transcripts were prepared using a clean transcription approach, which captures the content and meaning of spoken language while omitting fillers (e.g., “um,” “you know,”) to enhance readability, and applies consistent speaker identification, punctuation, and formatting to ensure clarity and uniformity. A range of transcription tools were used: where available, existing transcripts were lightly edited; shorter recordings were transcribed manually; and longer recordings were transcribed using Otter AI (Otter.ai, Inc), then manually reviewed and edited for consistency.

Gender, age, country, and sleep duration of each person with IH were recorded when explicitly mentioned. When not directly provided, gender was inferred from normative gender-conforming names or social media handles, age was estimated based on typical life events within Euro-American contexts (e.g., starting a job after university), and country was deduced from contextual clues within the content (e.g., local transit network names). Given the uncertainty of such inferences, demographic data were treated with caution and were not used as variables in the analysis.

### Data analysis

Transcripts and fieldnotes were manually analyzed by specialists in social sciences of medicine (AS, EB) using reflexive thematic analysis, which incorporates deductive and inductive reasoning [36]. Initial themes were identified based on prior research (deductive) and additional themes emerged from the data (inductive). Codes were iteratively refined to ensure they captured the full range of subject experiences.

The analysis involved familiarization with the data, coding, theme development, and theme refinement. The final themes were reviewed collaboratively by the analysts, with consensus reached on their organization into overarching categories and sub-themes using a tool called Miro (www.miro.com; formerly known as RealtimeBoard Inc.).

Concept saturation was assessed continuously during coding [37]. Saturation was reached when no new themes or insights emerged, and existing themes were fully explored. Saturation was confirmed by comparing new data with previously coded themes to ensure no further information was revealed. Although the frequency of theme mentions was recorded, the objective of this analysis was to describe the lived experience of IH; the study design was not appropriate to determine the prevalence or frequency of symptoms or impacts. Therefore, the frequency of mentions did not influence reporting.

The researchers maintained reflexivity throughout the analysis, meaning they actively reflected on how their own disciplinary backgrounds in the social sciences of medicine and healthcare might shape their interpretations and influence the research processes [38]. Regular discussions were held to identify and mitigate potential biases, and collaborative coding was used to improve analytical rigor. The team remained focused on faithfully representing the voices of PwIH, ensuring that emerging themes were grounded in the data rather than influenced by preconceived assumptions. Representative quotes were included to illustrate themes and findings.

### Ethical considerations

This study was deemed exempt from review by the WCG Clinical Institutional Review Board (wcgclinical.com). Data were collected from publicly accessible sources. No private groups, websites, or profiles requiring administrator approval were accessed. Fair use doctrine allows limited use of such material without permission for research purposes under US copyright statute [39]. The terms and conditions of each data source permitted the use of publicly available content for academic research, and all data were collected manually. Numbered identifiers were assigned to each poster, and personal information was removed from post transcripts.

## Results

### Search results

**Fig 1:**
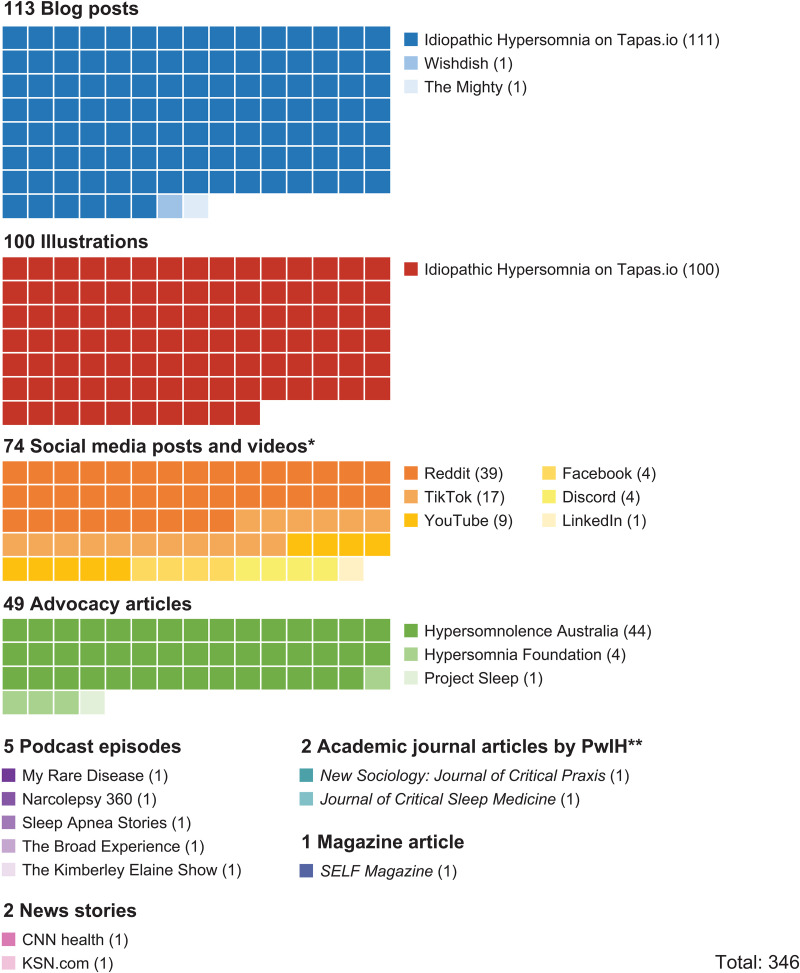
Included data sources. *Posts identified from Instagram and X/Twitter did not contain the depth and detail required for inclusion. **A self-ethnography by an anthropologist living with IH and a perspective on teleworking from an academic psychologist living with IH. IH, idiopathic hypersomnia.

A total of 346 online posts created by 123 PwIH were included. Over 90% of the content consisted of blog posts, advocacy articles, and social media posts or videos shared across various platforms (Fig 1). The media types included online text (214 posts), illustrations (100), audio (5), and video (28). Posts were created over a 10-year period, from November 2012 to October 2022.

Most people (87%) in the sample appeared to be female, based on information available for 62 PwIH. Ages ranged from approximately 16–60 years old, with most between 20 and 40 years of age. Most posts did not contain geographical information; those that did were from the United Kingdom, United States, Australia, Finland, Germany, and Canada. Seventeen individuals reported sleep duration, with 12 of these individuals (71%) sleeping ≥10 hours per 24 hours.

### Experiencing the symptoms of IH

The experience of living with IH symptoms was categorized into 10 major themes (Table 1).

**Table 1:** Symptom experience of idiopathic hypersomnia.

Theme	PwIH, n	Patient experience	Clinical definition
**Little time for anything but sleep**	23	Life is dominated by sleep; daily functional hours are extremely limited	Prolonged sleep duration: Total 24-h sleep time is ≥ 660 mins (typically 11–14 h) on 24-h PSG or by wrist actigraphy with a sleep log averaged over ≥7 days [3]
**Never feeling fully awake**	17	Never experiencing normal wakefulness; living between sleep and wake states	Not currently recognized
**Relentless and imperative sleepiness**	46	Persistent need to sleep that requires constant exertion to resist; often a futile battle	Excessive daytime sleepiness: Daily periods of irrepressible need to sleep or daytime lapses into drowsiness or sleep [3]
**Non-restorative sleep**	17	Sleep is not refreshing or restorative, leading to sensation of chronic sleep deprivation and difficulty regulating emotions	Non-restorative sleep: Subjective experience that sleep has not been sufficiently refreshing, often despite appearance of physiologically normal sleep [40]
**Extreme difficulty waking and getting up**	41	Extreme difficulty waking and getting up, with impaired speech, balance, cognition, dexterity, motor function, and irritability	Severe and prolonged sleep inertia or sleep drunkenness: Prolonged difficulty waking up with repeated returns to sleep, irritability, automatic behavior, and confusion [3]
**Acting without awareness or memory**	6	Speaking or acting without full awareness or control, with total or partial loss of memory	Automatic behavior: purposeful but inappropriate activity that occurs with the patient partially asleep [41]
**Microsleeps and long daytime naps**	20	Resisting sleep may lead to microsleeps; naps of unpredictable duration steal valuable time	Microsleep: Very short periods of sleep that can be measured in seconds. Long (>1 hour) unrefreshing naps
**Brain fog and other cognitive difficulties**	25	Multiple cognitive difficulties: “brain fog,” attention, memory, decision-making, impulse control, information processing	Cognitive symptoms that may or may not be linked with excessive sleepiness, related to an underlying neuronal dysfunction, which reduces concentration and impairs information processing, leading to a complaint of lack of clarity of mental thinking and awareness [42]
**Limited physical energy**	22	Energy is finite, easily depleted and must be carefully prioritized and rationed within and between days	Severe fatigue: Should be distinguished from sleepiness; may significantly add to the experienced burden of the disorder [3]
**Vivid dreams and hypnagogic hallucinations**	8	Vivid dreams can be intensely detailed and emotional. Hypnagogic hallucinations often combine visual, auditory, tactile, and kinetic phenomena. Both can be so realistic that they are difficult to distinguish from waking life.	Vivid dreams: Often bizarre, frightening, or complex in structure. Hypnagogic hallucinations: Often combine visual, auditory, and tactile phenomena [3]

PSG, polysomnography

#### Little time for anything but sleep.

PwIH described lives dominated by sleep. Sleep requirements varied, with many in our sample reporting they could not function on fewer than 10–18 hours of sleep per day. Work was frequently mentioned as a secondary priority, leaving “no time for anything else,” such as relationships and self-care.

*“When you need as much sleep as I do, working full time means you have no time for anything else. No time to cook or clean or look after yourself properly. No time to see family or friends and maintain healthy relationships. No time to enjoy life.*” [Person 15].

“*On a typical day I need to sleep about 15–16 hours. Try fitting that into 24 hours with an 11-hour shift at work, 1 hour of commuting, and needing to eat*.” [Person 4].

“*Imagine how you could do anything outside of your home if you needed to find a safe place to sleep [i.e., nap] twice a day, each day. How would you travel, how would you even run errands or join a friend for a matinee? Because of my sleep schedule and severe brain fog I only have a few hours each day in which to live my life*.” [Person 3].

“*I literally run out of wakefulness. When there are only 12 wakeful hours in a day and approximately 1/3 of this is consumed by my morning wake up routine (of sleep inertia), there isn’t much time for anything else*.” [Person 1].

“*My debts and responsibilities mean that after sleep, work is the priority in my life, and all else – my amazing wife and family, friendships, and everything else – gets barely a look in and the scraps of whatever attention and consciousness I can muster when not sleeping or working*.” [Person 12].

#### Never feeling fully awake.

PwIH reported that they never experience normal wakefulness.

“*At best I feel awake like a normal person for a few hours per year. It’s so rare, those times are clearly emblazoned in my memory*.” [Person 3].

“*I get it; we’ve all felt tired. But I have never felt awake*.” [Person 48].

They used terms like “no man’s land,” “underwater,” and “constant tug of war” to evoke the feeling of living between sleep and wake states, their brain held “hostage” or “prisoned by sleep” at all hours of the day. Every action requires tremendous effort and is accompanied by a sensation of sluggishness or incapacity, “*like wading through tar*” or “*walking through quicksand.*”

“*For me feeling tired is part of my every hour, it’s being awake but not aware. My eyes are open but my mind isn’t embracing my physical awakening, it’s as if my brain stays in no man’s land, between awake and asleep, opposing, warring states*.” [Person 61].

*“Living in and under water is my analogy for how it feels to live with hypersomnia. While most of the world sleep in shallow water at night and pop up onto the dry land where they stride about all day, I sleep in the depths and only make it to more shallow water during the day, still over my head though.”* [Person 37].

“*Another way to describe it is if you’re given anesthesia, but then you’re told you must stay awake. That’s how we feel all day*.” [Person 57].

#### Relentless and imperative sleepiness.

PwIH experience excessive daytime sleepiness as a “constant slow drag,” “heaviness,” or “venom” that “*is just slowly reaching out for you, and you have to constantly exert yourself like you’re tensing a muscle the whole day long just to really stay awake and focused and in-the-moment.*” [Person 19]. At times, this sleep pressure builds to the point of feeling like “an order,” “a demand,” or “a riptide or undertow […] that sucks me out to sea” [Person 37], forcing PwIH to sleep during the day.

“*Sleepiness […] is very difficult to get past. It’s the unquenchable need for sleep, you can fight it and you know it’s coming but there’s only so much you can do before it inevitably wins and your eyes flutter shut.*” [Person 61].

“*My brain is trying to tell my muscles to contract but they are unable to lift the weight. Gravity becomes an irresistible force, my body starts to collapse on itself, leaning on any surface, vertical or horizontal until I finally settle in my default complete horizontal position*.” [Person 4].

“*The feeling of sleepiness becomes so strong that I literally feel like I can’t go on living, so my entire world revolves around this sleep need.*” [Person 3].

*“I have to literally push myself through every day. I fall asleep on the bus to work. I fall asleep at my desk. I have trouble concentrating. I have difficulty with my memory. The fatigue, the brain fog, the constant need for sleep is relentless. I never get any reprieve.*” [Person 25].

Some try to stay awake by engaging in continuous activity.

“*My family often tell me to slow down; that I don’t always have to be on the move. Unfortunately for me, being on the move is what keeps me awake*.” [Person 69].

#### Non-restorative sleep.

PwIH reported that their sleep is never restful, refreshing or restorative, regardless of how much they get. Consequently, PwIH live with a sensation of chronic and irreparable sleep deprivation, which is emotionally and cognitively draining.

“*No matter how much sleep I get, I never feel rested...I am constantly in a state of sleepiness. I never feel fully awake. I am fatigued and have a foggy brain, I can’t think straight, and I become confused quite easily as I feel so sleep deprived. Although technically I’m not sleep deprived because I get more than enough good quality sleep. Sleep just never, ever, leaves me feeling refreshed, no matter how much I have*.” [Person 1].

“*People with IH can sleep hours upon hours, but we don’t get restorative, restful sleep. Therefore, we are constantly exhausted*.” [Person 47].

People in our sample thought that this is an important but misunderstood part of the disorder.

“*This is one of the things that is so important for people (including doctors) to understand […] People with IH do not have the benefit of sleep […] All I want and need is sleep, and yet sleeping never makes it feel any better. I’m constantly exhausted!*” [Person 25].

#### Extreme difficulty waking and getting up.

PwIH described the extreme difficulty of waking and getting up every morning, frequently referred to as sleep inertia, as “a feat of Olympic effort,” “an enormous battle,” “absolute torture,” and “almost physically painful.” People in our sample reported that they sleep through fire alarms, need to be shaken awake, use alarms for the hearing impaired, or construct “exceedingly elaborate alarm ritual” and obstacle courses to avoid falling back asleep. Sleep inertia can last for hours and reoccur after naps. This debilitating state impairs motor function, dexterity, and balance, while causing cognitive issues such as speech and language, executive function, and memory challenges.

*“Often I cannot engage in any type of conversation as I feel confused, disorientated and need this time to get my bearings […] My brain and body are slow to respond and I have to sit up on the bed for a few minutes before I can try to stand. Sometimes I will fall back to sleep for a while, most times I will attempt to stand and then fall back on the bed, but if I get up and going, then momentum takes me crashing to the bathroom staggering and hanging onto walls before I collapse on the toilet –where I will often lay my head on the sink and sleep again. This is called ‘sleep drunkenness’ and also extends to my inability to properly speak or think in the morning, or to use my hands.”* [Person 12].

*“It is difficult to sustain an awake state long enough to act on the intention of arising. This is when I especially feel that I am drowning in sleep—I just keep slipping back under the surface.”* [Person 37].

*“I cannot think properly in this time and make poor decisions. My speech is affected. My balance and spatial awareness are affected and I will bump into walls, furniture, and doorways.”* [Person 15].

#### Acting without awareness or memory.

PwIH often experience automatic behavior, which was described as “*performing a task or tasks without later having any memory of you actually doing it. Your body moves and does things while your brain is offline.”* [Person 2]. Common examples were turning off the alarm clock, misplacing things, or saying “things you don’t even remember later.” Some PwIH reported more serious or dangerous behaviors, for example, while driving, mainly when undiagnosed.

“*But automatic behavior can be dangerous […] not all of my automatic behavior[s] are ‘silly mistakes’ […] I have left pots on my stove until the bottom has burnt out and the fire alarm goes off. I have left my daughter in her car seat too... I don’t even want to relive that story.*” [Person 10].

“*I remember passing junction 7 of the [highway]. And the next thing I remember is being at junction 9. So for two junctions part of my brain was awake and part was asleep*.” [Person 21].

#### Microsleeps and long daytime naps.

PwIH described two types of daytime sleep experiences. One is a trance-like state called a “microsleep,” in which PwIH may appear awake, but “*It is a stage where part of my brain has shut down and I literally blank out for a minute or two...or longer. I can’t understand a word you say despite trying the best I can.*” [Person 2]. Microsleeps are more likely to occur when performing monotonous or sedentary activities and when resisting the pressing sleepiness of IH.

“*Day-to-day living with IH means that you get used to falling asleep in public […] and you’ll often go into a ‘trance-like-state’ falling into microsleeps while attempting to read/do sedentary activities*.” [Person 7].

The other sleep type is daytime naps. When PwIH are compelled to sleep during the day, they are unable to predict or control nap duration, using metaphors like “nap roulette” to describe these episodes. One person with IH shared an example where they had napped for 8 hours [*Person 90*]*.* The experience of losing an hour or more to sleep is frustrating and disorientating, like “*time travel, not the fun kind*.” [Person 2].

*“Nap Roulette is the act of taking a nap and not knowing how long it will be. Will it be twenty minutes? Two hours? Will I wake up refreshed and ready to continue my day, or will I be even more exhausted and need to keep sleeping? I never know, but I spin the Nap Roulette wheel at least once a day.”* [Person 31].

#### “Brain fog” and other cognitive difficulties.

PwIH experience several cognitive difficulties, including “immense brain fog, which hinders my ability to think clearly” [Person 48], problems with sustained attention or focus, poor memory, impaired decision-making and impulse control, and speech and language processing issues.

“*I often lose my train of thought in the middle of a conversation. I don’t mean once in a blue moon, I mean almost every single conversation I have. I can be looking right at you and listening to what you say, and immediately forget everything you just said, like it completely failed to absorb. I find it really hard to absorb audio information. I can’t watch video without subtitles, despite having perfect hearing*.” [Person 9].

*“I constantly have to ask others to repeat themselves when telling me how to spell their name, email address or giving me their contact numbers as my brain has trouble taking in and retaining the information.”* [Person 52].

These experiences were often discussed alongside sleepiness, non-restorative sleep, sleep inertia, microsleeps, and automatic behavior; the extent to which cognitive difficulties represent distinct symptom phenomena was unclear.

“*I become confused quite easily as I feel so sleep deprived.” [Person 1].*

*“Brain fog has my brain locked up. I know that information is there somewhere in my brain, but it is too sleepy to access it*.” [Person 18].

“*They say that memory is the ghost of attention, so whatever you’re able to focus on in the moment is what you’ll be able to remember, but if you’re so foggy and tired you can’t focus on anything then of course you just kind of lose all of these memories […] large periods of my life that are just kind of foggy in that way*.” [Person 19].

#### Limited physical energy.

PwIH discussed physical energy as a finite resource that is rapidly depleted and must be carefully managed. They often used the words “fatigued” and “exhausted” and distinguished these terms from “tired” and “sleepy”.

*“Terminology is really important. My sleep specialist explained that tired, fatigued, and sleepy mean really different things. To be tired is to feel ‘can’t be bothered’ or have had enough mentally. Fatigue is when your body physically has had enough like after a hard day’s work and you need to rest. Sleepy is when you feel as though you just want to close your eyes and go to sleep. You can be tired without being fatigued, fatigued without being sleepy, and sleepy without being tired. (Or you can be all three at once!)”* [Person 32].

*“There are distinct differences between these words that are often used interchangeably by others. I think perhaps the various meanings of these are so distinct to me because I live my life in a perpetual balance of the three [...] Having this disorder has changed the way I look at words and how I interpret them. In my view one can be tired, yet not sleepy. You can be sleepy yet not exhausted. You can be exhausted but not sleepy.”* [Person 61].

PwIH “become very fatigued very quickly,” “attempt to complete tasks slowly with regular rest breaks,” and ration their energy expenditure throughout the day and between days.

“*Most days I have so little energy, that I have to choose where to spend it. That means often having to forgo things that I need, like home cooked meals, household chores, or even showering. Things that take a lot of physical energy that we take for granted.*” [Person 9].

“*I physically do not have the energy to clean, do laundry, brush my teeth, shower, make food. I have to rest in the process of typing a single sentence*.” [Person 41].

“*No doubt any father would love to have the energy of their kindergartener, but I’m sure most are not wanting for sufficient physical capacity to give their kids just 15 minutes of their time*.” [Person 38].

#### Vivid dreams and hypnagogic hallucinations.

PwIH experience vivid dreams and hypnagogic hallucinations, which can be “distressing” or “terrifying”. People in our sample described false awakenings, which are dreams in which the sleeper believes they are awake when they are still asleep, and dream-reality confusion, where one has difficulty or is unable to determine whether an event or experience occurred during waking or dreaming. They also reported early-onset REM sleep phenomena such as vivid visual, auditory, tactile, or kinetic hallucinations when falling asleep and waking up, and dreaming during naps. Sleep paralysis was infrequently discussed.

“*One day in class while struggling to stay awake I looked down and suddenly my desk was floating on an island in the middle of the ocean*.” [Person 18].

“*I’ll have a like, dreamlike experience where I’ve woken up, I’ve brushed my teeth, I’ve had a shower, I’ve had breakfast, put clothes on, gotten ready for work. And then I wake up and realize I’ve been asleep the whole time. And then now I’m running late, ‘cause you know, I thought I was awake*.” [Person 63].

“*Sometimes I don’t know whether something happened in my dreams or was real*.” [Person 50].

### The impact of IH

**Fig 2:**
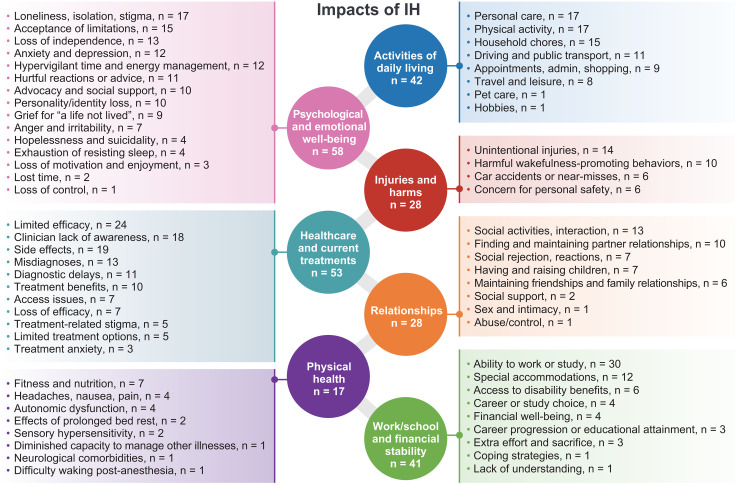
Impacts of idiopathic hypersomnia. n, number of PwIH in our sample referring to each impact. Each PwIH could be included in more than one sub-domain for each impact domain.

Descriptions of the impact of IH were categorized into seven domains, each with subdomains describing specific impacts (Fig 2).

#### Psychological and emotional well-being.

PwIH must constantly balance present activities against future energy and wakefulness requirements. To achieve this, they carefully control their time and environment; unpredictable situations threaten their ability to function. Living in a state of chronic hypervigilance is stressful and anxiety-inducing.

“*The present moment must often be measured against the moment to come: if I go to this talk now, I will be too tired for that class later.*” [Person 49 citing Kafer 2013 [43]].

“*I can’t do things outside my schedule. My plans are concrete. They have to be if I want to survive*.” [Person 2].

“*The window of ‘wakefulness’ on a better day is small. I find that if I have been awake for around two hours, I rapidly decline back into that sleep inertia feeling. This can be fairly problematic for example if I am out and waiting for a specialist appointment and the doctor is running late. By that stage, I am already dozing in my chair waiting for him and become emotional with sleep deprivation by the time I am called. I need someone to drive me home*.” [Person 34].

Many PwIH in our sample rely on family or friends for assistance with waking up, personal care, daily activities, driving, and financial support. They express frustration at their loss of independence and “*often struggle with guilt for relying on [my partner] so heavily*” [Person 11]. Further, living with such a “poorly understood, hidden illness” that is widely misunderstood, trivialized, disbelieved, and invalidated is profoundly lonely and deeply isolating.

“*Even the people who want to understand, don’t really understand. Like my mother […] But how do you tell a healthy person how IH feels?*” [Person 20].

“*[Healthy people say things like]’I’m tired too honey, believe me’ ‘You just need to exercise more. Exercising gives you energy!’, ‘Are you eating right? These foods give you a natural energy boost!’,’ ‘You just need to drink more water!’, ‘It must be stress related, try meditating!,’ ‘If you’re really that tired, just take a short 20-minute nap. You’ll feel much better after!*’” [Person 36].

“*I’m looked at as if my exhaustion is just a matter of will power or a lack of motivation*.” [Person 47].

PwIH feel that the condition has robbed them of their lives and grieve that they are “*watching the years go by in a blurry fog […] gutted at the realization I am sleeping my life away.*” [Person 12]. Many struggle to maintain a sense of identity and purpose amidst the loss of careers, hobbies, and relationships.

“*IH transformed me from a hardworking doctor with a full family and social life into a nearly homebound shadow of my former self*.” [Person 3].

“*I’m trying to grasp that thread that connects me to my old self and to what I love to do and hold it close, because I know what makes me a person - what makes me ‘me’ - is almost gone*.” [Person 22 citing Klentzman 2021 [44]].

PwIH express a loss of vitality and enjoyment of life, as well as profound hopelessness and suicidality.

“*Your will to do things just kinda dies, [… and] then you’re always trying and trying again. It just gets worse. You kinda die inside from being tired all the time.*” [Person 92].

“*It has taken everything from me. I no longer enjoy life. […] Life isn’t worth it like this*.” [Person 75].

“*It is a testament both to an excellent therapist and my immense willpower that I learned to cope with IH well enough to want to remain alive. I do not say that lightly*.” [Person 3].

“‘*In Memoriam of those who slept away and ended their lives’ is what I imagine would read in a memorial for those who were beaten down by IH and developed depression, anxiety, and an endless hopelessness caused by its many symptoms. I imagine a headboard of a bed as the body of the memorial because those lost were bound to their beds. They were bound to sleep*.” [Person 2].

PwIH find that their relentless sleepiness can manifest in anger and irritation:

“*I could sometimes unfortunately be short-tempered with my husband because it feels like you’re carrying such a burden that [the] slightest thing put on top of that is an intolerable imposition.*” [Person 19].

“*I was at the stage where I would get so thoroughly exhausted that I felt physically ill and could literally not function anymore unless I slept. It was debilitating. I was getting more and more irritable, snapping at my family, getting angry and upset over trivial things*.” [Person 35].

#### Activities of daily living.

PwIH must trade their excessive need for sleep and limited energy against everyday activities such as bathing, grooming, household chores. Sleep inertia introduces additional personal care and mobility challenges upon waking, while many report regularly skipping meals due to an overwhelming urge to sleep, difficulty waking, or lacking the energy to grocery shop.

“*I set my baseline minimum for a successful day at feeding myself - that’s it. No showering, no getting dressed, no washing the dishes, no paying the bills, no calling a friend. I can’t predictably do any of these things*.” [Person 3]

“*[Because of impaired grip strength due to sleep inertia] I have to get someone else to open the juice bottle for me so I can take my pills*.” [Person 43].

“*At times I’ve had to have help with simple everyday tasks such as cleaning, cooking and even driving. I sat in my car parked at the grocery store crying for half an hour just trying to find the energy to go inside. Some days I honestly feel like I’m too tired to breathe*.” [Person 18].

PwIH often struggle to find the time and energy to exercise. Some experience post-exertional malaise.

“*I cannot exercise – I simply do not have the time or the energy*.” [Person 18].

“*I used to love dancing and hiking and rock climbing. I used to enjoy going for long walks, and even going to the gym. Now, I find it hard to get out of bed*.” [Person 9].

“*[Exercise]* c*an cause an immediate crash after/give a bout of lethargy that can last [anywhere] from hours to days/make the sleepiness so bad that nothing else than recovery can be done for a long while.*” [Person 2].

Brain fog and memory issues can interfere with activities like personal administration, remembering medical appointments and recalling their content.

“*I cannot remember appointments despite having written them down in multiple places. I can’t rely on others to remind me of everything.*” [Person 18].

“[*I] cannot remember the conversation I just had with the respective medical professional as soon as I leave their room.*” [Person 34].

“*I’ve signed documents that I probably shouldn’t have signed, or [made] bank transfers that were incorrect.*” [Person 22].

“*Not only did I not realize I had left my purse somewhere, I did not remember going to [the store] at all that week*.” [Person 52].

Driving and public transportation pose significant challenges for PwIH. They describe developing “behavioral strategies to ensure safety for myself and others when on the road” [Person 54], carefully timing trips to coincide with peak periods of wakefulness and limiting distances.

“*If I had to drive longer than 30 minutes it was a constant battle of ‘do I keep splashing water on my face and blasting cold air to push through till I’m home, or pull over and take a nap for 45 minutes to 2 hours?*’” [Person 71].

“*Public transport is difficult for me to use unaccompanied as I may fall asleep which places my safety at risk. I also forget to tag off my [bus pass] as my cognitive abilities wane after being awake for more than two hours and I have received a number of fines*.” [Person 15].

“*Using a bus alone is hell for me […] Every blink can lead to me falling asleep in public (again) and missing my stop … Every brainfoggy thought can lead me astray...and every bus has always people who give me dirty looks for nodding off*.” [Person 2].

“*I’d love to go to a party, a concert or even the movies with friends but I can’t drive far for fear of falling asleep at the wheel.*” [Person 29].

PwIH found traveling away from home challenging, as it is difficult to prepare for trips and fully participate in activities.

“*I went on a 3-week family holiday driving to the middle of Australia and back and recall them jokingly commenting, ‘[Person’s name] have you seen any of the amazing sights...you have been asleep the entire trip,’ and they were right. I would sleep for 10 hours at night and in the daytime, I would fall asleep when I wasn’t driving.*” [Person 54].

#### Injuries and harms.

Sleep inertia causes impaired cognitive and motor function, which can make it difficult to stand, walk, grasp, and speak for several hours after waking, increasing the risk of household accidents. People in our sample described tripping or walking into walls and furniture, falling downstairs, and spilling hot drinks in this state.

“*I have objects placed to hang on to, to make it to the bathroom, hopefully unbruised*.” [Person 5].

Cooking can be a hazardous activity due to falling asleep and automatic behavior.

“*I have accidentally burnt and cut myself numerous times.*” [Person 34].

“*A friend with IH [...] almost burnt down her kitchen twice by falling asleep*.” [Person 21].

“*I’ve fallen asleep with food on the stove. Woke up to burning smell, more than once*.” [Person 70].

People in our sample also described near-drownings, sunburns, and machinery accidents due to falling asleep or impaired alertness.

“*I’ve had sunburns from falling asleep outside in the blazing sun*.” [Person 71].

“*I have almost drowned so many times […] I am no longer allowed near water without a life vest*.” [Person 51].

Sleepiness, brain fog, microsleeps, and automatic behavior pose driving risks, leading to near-misses or accidents. Falling asleep in public is another safety concern for PwIH, who fear assault or theft while at their most vulnerable. To combat sleepiness, some PwIH use potentially harmful behaviors like overhydration, fasting, and self-inflicted pain.

“*Before being diagnosed I used to drink a lot of water in college because I hoped wanting to pee would help keeping me awake during lecture.... That and not eating enough and pinching myself...The amount of discomfort and unhealthy things done to fight against something unknown!*” [Person 23].

*“I lived in a forest as a kid but went to study [in a] close by city and the amount of times I missed my bus stop for falling asleep and ended [up] in strange places...was almost weekly thing. I slept in park benches in the city, and we have a massive drug problem here...it is [a] straight up miracle that I never got mugged, or kidnapped.*” [Person 2].

“*Sometimes I’ll intentionally avoid eating to stay more awake/ functional […] When disciplined, I’ll push meals to the end of the day to increase productivity and help sleeping but I’m not doing this every single day. […] Today I’d only fasted 25 hours, but I’ve fasted for weeks before.*” [Person 79].

#### Relationships.

IH profoundly impacts social functioning, relationships, and family life. The constant struggle with sleepiness, fatigue, and time limitations makes it difficult to fulfill social commitments, engage in leisure activities, and participate in social events. Difficulty concentrating, memory lapses, and mood instability further impact relationships and social interactions. Negative reactions and misunderstandings from loved ones contribute to strained relationships and exacerbate feelings of loneliness and isolation.

“*We get called flaky for not showing up...but what they don’t see is that we wanted it so much...but as we were getting ready, our bodies gave up on us like cars that leave you on the side of the road. We get called ignorant when we get there...but they miss how hard it was to get there […] We showed up...and for them, it isn’t enough. We get called boring because sometimes we can’t even speak from exhaustion. Our cognitive abilities drop to mumbling and keeping it together is all we can do. And more often than not...people see these things and decide that they don’t want to invite us anymore*.” [Person 2].

*“It’s hard to make and keep friends when people think you’re not interested in them or what they’re saying. But it’s not true - I am interested. I just have difficulty concentrating on what’s being said and following the story.”* [Person 29].

*“[My mom] really tries to accept my disorder, but it’s hard for her. She sometimes gets really angry at me, if I can’t get up*.” [Person 20].

IH also significantly impacts romantic relationships, hindering people’s ability to meet and spend time with partners.

“*And then there’s dating...my time and energy today simply does not exist, if dating did happen how’s the relationship to happen? [...] IH affects not only my everyday life but my entire future.*” [Person 18].

Many discussed the impact of irritability and cognitive issues on relationships:

“*IH makes me emotionally labile, irritable and adds enormous stress to my relationship with my significant other*.” [Person 3].

“*This inability to be able to fully focus on the task at hand or a conversation is definitely an issue my wife has noticed in our personal lives […] She will often have to repeat things that were discussed previously as I have forgotten the conversation or was unable to focus in the first place due to being half asleep*.” [Person 12].

While some people in our sample had supportive partners, others attributed separations to IH. Parents with IH discussed significant struggles managing childcare alongside their condition, while others made the difficult decision not to have children due to their condition*.*

“*When I did have two children, I realized that this was a whole new world of hell [...] I was not going to be able to continue to work and have children and do everything else that’s expected of people with what was happening with me*.” [Person 22].

“*I even had to make the heartbreaking decision not to have children because I’m too sick to care for them*.” [Person 3].

“*Even without children my partner would have to take on much of the home responsibilities*.” [Person 18].

“*I am fearful that I might never be able to have a family. I am happily married, and my husband would like children one day, however as it stands, I am overwhelmed by the thought of how tired and incapable I might become with the inevitable sleep deprivation that comes with having children, and what that could possibly do to our relationship.*” [Person 7].

#### Work/school and financial stability.

PwIH find it challenging to synchronize their limited hours of wakefulness with expected rhythms of work and school life, while traveling to a place of work or study can also be difficult. Some PwIH work or study full-time, particularly if they can control their schedule or obtain accommodations (e.g., later start times, working from home, asynchronous hours, online courses, scheduled naps)*.*

*“Difficulties including fatigue and impaired concentration may result in a later working pattern and working outside of contracted hours to complete tasks and compensate for time lost during the day, consequently reducing time for personal activities, particularly in long-sleeping individuals with IH. The long-term experience of playing catch-up may serve to perpetuate the onset of, or pre-existing, worry […] and rumination […] about work performance, which ultimately may facilitate overworking and possible burn-out in those with IH.”* [Person 101 [45]].

*“Whenever I had a gap between clients of at least 2 hours I would drive back home, set my alarm, nap, then go back to work.”* [Person 54].

*“I had my driving license withdrawn […] My world stopped […] now I couldn’t do the one thing I loved, my job […] Initially, for 2 days, I tried walking to work...it took 2 hours each way. Realistically I could never keep that up […] Losing my job really broke me. I couldn’t see how I was ever going to get my life back.”* [Person 13].

For those who do have full-time work or study, it carries a large personal cost, and many PwIH experience discrimination and moralizing judgments that impede professional and academic success.

“*There are some amazing people [with IH] I’ve talked to worldwide that do work in very demanding jobs. But they spent all of their spare time asleep*.” [Person 21].

“*I’ve almost certainly hit my career ceiling before reaching 30 years of age as it is just not possible to progress any further with the actual limitations on my concentration and prejudices relating to perceived effort/dedication*.” [Person 38].

Some PwIH must reduce their hours or are unable to work or study. They describe a resulting loss of identity, self-worth, and financial stability, compounded by extreme difficulty in accessing disability benefits.

“*IH is not a disability that is recognized by the NDIS [Australian National Disability Insurance System] and the medical community does not seem to understand how disabling this disorder is. I do not have the wakefulness or energy to try and obtain the reports needed to access the NDIS when it means I have to try and explain everything to my doctors about how IH affects me every day. If I had a well understood disorder the need for these supports would not need to be explained. Focusing on the difficulties of my disability is emotionally draining and having to explain time and again how it impacts me is exhausting*.” [Person 15].

#### Physical health.

PwIH often struggle to maintain their fitness and nutrition due to a “*mix of low energy, not being able to exercise, and having to eat cheap and easy to prepare foods*” [Person 9] and worry about the long-term health consequences. Managing other health concerns is made more challenging by the overwhelming day-to-day burden of IH.

“*IH also puts you at risk of other conditions caused by malnourishment and/or dehydration due to sleeping through meals, lack of exercise or being in a horizontal position for so long each day, or just the general effect of having a “more pressing” health condition to worry about making other medical problems only urgent when they are at a worryingly late stage*.” [Person 11].

If PwIH do not get enough sleep, they “*feel physically sick with headaches and extreme nausea*” [Person 18]. At the same time, prolonged bed rest can result in additional health challenges.

“*Research has shown that the effects of prolonged bedrest due to excessive sleep […] and even the excessive sleep itself can be more harmful than not getting enough sleep.*” [Person 22].

“*I even have aches and pains simply from sleeping so much and being in one position for such a long period of time*.” [Person 18].

Some PwIH experience symptoms of autonomic nervous system dysfunction, such as high heart rate, orthostatic hypotension, and heat sensitivity, which adversely impact daily life. People in our sample also described “*significant sensory issues relating to touch, sight, sound and smell, and become particularly agitated when in crowded or noisy spaces*.*”* [Person 34].

“*When I’m standing my heart rate it gets extremely high[,] basically my autonomic system is all out of whack and my body doesn’t really adjust very well to changes*.” [Person 19].

“*We can’t simply stand higher temperatures well, and are more likely to get heat exhaustion and even heat stroke*.” [Person 2].

#### Healthcare and current treatments.

*“Getting a diagnosis for IH is HARD”* [Person 2], involving numerous clinical visits, diagnostic tests, and misdiagnoses such as depression, chronic fatigue syndrome, hormonal issues, connective tissue disease, attention deficit hyperactivity disorder, obesity, thyroid disease, and anemia. Many recounted their IH symptoms being dismissed as life-stage–related fatigue associated with growth spurts, adolescence, studying, working, parenthood, and middle-age, and PwIH frequently endure dismissive attitudes and unhelpful suggestions from healthcare professionals, prolonging access to a diagnosis.

“*Within the space of a year, I probably saw my general practitioner 10 or 11 times. Each time, I was made to feel like I was wasting his time, and I would leave his office with a different suggestion.*” [Person 30].

Gender and weight discrimination also delay diagnosis:

“*I’ve been tested for apnea three times actually because I’m also overweight. And being an overweight woman in the American medical system means you have a tendency for doctors to discount what you’re telling them*.” [Person 98].

Often a frightening driving experience is required to get “*anyone to pay attention to how I was feeling*” [Person 13] or to lead PwIH to insist on diagnosis or referral.

Current treatments impose additional burdens on PwIH. Off-label stimulants and wake-promoting agents approved for use in narcolepsy were the most frequently discussed medications in our sample. Although these medications can partially and temporarily reduce feelings of excessive daytime sleepiness, PwIH reported lack of efficacy or loss of efficacy over time and unpleasant side effects like appetite suppression, intensification of brain fog and concentration difficulties, hair loss, tics and twitches, nausea, and panic attacks. This is a “*no win situation*” [Person 22]. Additionally, sleep inertia makes it very challenging to take medication in the morning or as prescribed.

“*It never really resolved my sleepiness. It was like my body was awake, but no-one was home. My dose was gradually brought up to 40 mg, at which point my heart rate would increase dramatically, with little in the way of symptom relief […] I would desperately need to nap, but my brain would refuse to turn off. This caused me to become irritable and angry at the tiniest things*.*”* [Person 9].

“*If I don’t take the meds, I’ll sleep my life away and want to die OR if I do I take the meds, I’ll have terrible side-effects and then also want to die*.*”* [Person 75].

“*[Medication] can sometimes actually make our situation worse! […] how helpful is keeping someone awake who has a very real need for excessive amounts of sleep? What do you think happens to someone who is kept awake when their brain needs sleep?*” [Person 22].

Some PwIH mentioned oxybate more positively: In one TikTok video, a young woman holds an amber pill bottle full of liquid to her lips as though about to kiss it – an ode *“To the med that’s thousands of $$ but is the only thing keeping me from (skull and crossbones emoji) myself.*” [Person 75].

Financial barriers, including high out-of-pocket costs and insurance coverage restrictions, can also hinder access to medications. Stigma associated with stimulants and oxybates further exacerbates the challenges of seeking and accessing treatment. For example, after an article in the *New York Times* [34] drew parallels between the use of gamma-hydroxybutyrate in Xywav and common “date rape” drugs, one person in our sample remarked that they have become reluctant to ask for these medications.

“*[We were] afraid to ask our doctors about different treatments or to increase dosages for fear of coming off as a drug-seeker. I’ve been biting my tongue for over a year about my meds which aren’t getting me through the day. If I was nervous to discuss Xywav with my doctor before, I’m absolutely terrified now. It might take another year for me to find the courage to even tell my doctor that my current meds aren’t enough.*” [Person 24].

“*My primary care physician was very uncomfortable with prescribing this controlled medication. I had to get a new prescription every 15 days, not just every month […] every 15 days and I would have to drive down physically, collect the prescription in paper form, and then take it to the pharmacy – it was kind of a nightmare*.” [Person 19].

## Discussion

This study provides new insights into the lived experience of IH, revealing its profound impact on every aspect of life, from daily functioning to major life choices and overall well-being. People in our sample shared complementary and convergent narratives that facilitated the creation of a thematic framework for understanding and communicating the symptom experience and impacts of IH. Such testimonies help legitimize a condition that is often misunderstood and trivialized, and provide a unique lens through which to appreciate its severity and complexity. Direct quotations from PwIH offer insights that cannot be captured in surveys, and allow clinicians, researchers, and policy makers to better grasp the critical need for increased awareness and more effective therapies.

This study complements and builds upon findings from the Sleep Consortium “Illuminate Hypersomnia” initiative, comprising a global survey of patients from 21 countries and an associated meeting held in April 2024 [25], which described the lived experience of persons with IH and highlighted the significant challenges faced by those living with the disorder. Together these studies provide essential context for informing future research, regulatory guidance, drug development, clinical practice, and public health discourse.

The thematic findings from this study provide important refinements to current clinical understanding of IH symptoms. For example, an important symptom experienced by PwIH is the feeling of never being fully awake, which was noted by Billiard et al (1994) [46] but is not currently clinically recognized. Sleep inertia is acknowledged in clinical diagnostic criteria; however, current definitions fail to capture the profound impairment it causes, including significant difficulties with speech, balance, dexterity, and motor function, which makes movement challenging and often necessitates assistance with daily activities. Similarly, while non-restorative sleep is clinically recognized, current definitions overlook the associated sensations of sleep deprivation, emotional dysregulation, and hopelessness that stem from the knowledge that no amount of sleep will alleviate the exhaustion. Clinical descriptions of excessive daytime sleepiness reflect the episodic, irresistible sleepiness that is characteristic of narcolepsy but do not adequately describe the relentless struggle to stay awake throughout the day experienced by PwIH. This distinction reveals the multidimensional nature of sleepiness and suggests that traditional assessments (e.g., the Epworth Sleepiness Scale) may not accurately measure its severity in PwIH. While diagnostic criteria for prolonged sleep refer to nocturnal sleep duration, PwIH emphasize their limited hours of daytime wakefulness, shifting the clinical focus of this key symptom. Lastly, delayed sleep phase, evening chronotype, and increased sleep times on weekends and holidays have previously been described in PwIH [47]. People in our study discussed these features in the context of sleep inertia, work schedules, and social roles, but they did not emerge as separate themes, possibly due to sampling limitations.

The impact of IH extends beyond the scope of generic quality-of-life instruments, which do not capture concepts like lost time, independence, life choices, self-actualization, financial well-being, work and school advancement, healthcare experiences, sleepiness, and fatigue. Consequently, current tools may vastly underestimate the true burden of this disease. Additionally, while healthcare costs may arise from injuries, car accidents, suicidality, comorbidities, and long-term negative health outcomes, the most significant burden falls on PwIH, who bear the costs and consequences of truncated education, reduced or abandoned careers, missed opportunities for relationships and parenthood, and more. Recognizing the extensive personal and societal costs of IH is essential for payers and policymakers to prioritize resources and support effective treatments.

Cognitive symptoms, limited energy, and excessive sleep inertia are central to the experience of IH, yet these and other symptoms are not routinely evaluated in clinical practice in favor of reducing excessive daytime sleepiness, improving the accuracy of diagnosis, and reducing adverse events [48]. As a result, many clinicians do not appreciate the far-reaching impacts of this disorder across social, psychological, and economic domains. The results from this study can help inform clinicians on the true burden experienced by PwIH, and help improve the dialogue to reduce stigma, align on treatment goals and expectations, and monitor progress. Results from this study can be used to inform the development of validated patient-reported outcomes and the design of robust clinical trials with patient-centered outcomes, with the goal of improving patient care and potentially new advances in drug development.

This study also demonstrates the value of SGOPE data as a valuable resource for exploring under-researched conditions like IH. Unlike traditional interviews, this method captures experiences that are important to PwIH using terms and concepts that are unbiased by researcher prompts or behaviors, and may contain insights that people hesitate to disclose in conventional research settings [49,50]. This is particularly true for PwIH, who expressed reluctance to openly share their experiences with clinicians due to pervasive stigma and negative experiences. By manually collecting and analyzing content, we gathered all types of unstructured data (text, illustrations, audio, video) from a variety of online platforms (e.g., patient advocacy blogs, news channels, social media). This approach facilitated comprehensive exploration of our research questions, drawing from a range of voices spanning different ages, countries, and levels of digital proficiency. Online content analysis is also a relatively quick and cost-effective way to gather patient-experience data from a geographically diverse sample of patients, particularly those that challenge traditional methods due to small populations.

A limitation of using SGOPE content for this type of research is that diagnoses were self-reported, with no way to verify demographic characteristics or clinical history. It is also difficult to determine the representativeness of these data, as this method is biased towards those who engage in online conversations about IH, and the underlying selection process is difficult if not impossible to quantify. This sample was skewed towards PwIH who are female and younger (20–40 years of age), which may omit important perspectives. Only English-language posts were included, which limits the cross-cultural relevance of our findings. Because online users can freely create, edit, and delete content, SGOPE data are ephemeral and dynamic, which limits the reproducibility of this study. Additionally, this methodology does not allow probing to seek clarification on the views expressed, and there is a risk that researchers may misjudge the meaning of personal experiences described. We did not engage PwIH as partners in the research process or validate these findings with patient groups; this engagement is critical for future research.

Using direct quotations from online content carries important ethical considerations [33]. While we followed best practices by utilizing only publicly shared content and removing identifying details, it is possible that original sources could be found via search engines. We considered alternatives to anonymization, like generating synthetic quotations or omitting them altogether, but chose to report data verbatim for several reasons. First, many people in our sample created content with the intention of advocating for PwIH or in hope of reaching a healthcare audience; directly sharing these perspectives helps amplify their original purpose. Second, PwIH used such vivid language and powerful imagery that reporting anything other than their words would diminish the poignancy and insight of their narratives. Lastly, research indicates most patients are willing to share social media data if it contributes to improving their condition and that of their peers [51]. Facilitating such improvements is the intention of this study.

Future research should build on these findings through in-depth qualitative interviews with people who have a confirmed diagnosis of IH. A discussion guide for these interviews should be informed by firsthand perspectives captured in this study, ensuring that the questions reflect experiences and priorities identified by people living with the condition. Diverse sampling across age, race, sex, gender, geography, socioeconomic groups, and life-stages is essential. If intended to support research and drug development, interviews should elicit and map patient-perceived relationships between symptoms, life impacts, coping strategies, and mitigating variables, and explore patient-defined treatment goals. Engaging patients and advocacy agencies as research partners will be critical to ensure that resulting interpretations and conceptual models remain aligned with patient perspectives.
